# The International Scientific Association for Probiotics and Prebiotics (ISAPP) consensus statement on fermented foods

**DOI:** 10.1038/s41575-020-00390-5

**Published:** 2021-01-04

**Authors:** Maria L. Marco, Mary Ellen Sanders, Michael Gänzle, Marie Claire Arrieta, Paul D. Cotter, Luc De Vuyst, Colin Hill, Wilhelm Holzapfel, Sarah Lebeer, Dan Merenstein, Gregor Reid, Benjamin E. Wolfe, Robert Hutkins

**Affiliations:** 1 Department of Food Science and Technology, University of California-Davis, Davis, CA USA; 2 International Scientific Association for Probiotics and Prebiotics, Centennial, CO USA; 3 University of Alberta, Department of Agricultural, Food and Nutritional Science, Edmonton, Canada; 4 Department of Physiology and Pharmacology, International Microbiome Center, University of Calgary, Calgary, Canada; 5 Teagasc Food Research Centre, Moorepark, Ireland; 6 APC Microbiome Ireland, University College Cork, Cork, Ireland; 7 VistaMilk, Cork, Ireland; 8 Research Group of Industrial Microbiology and Food Biotechnology, Faculty of Sciences and Bioengineering Sciences, Vrije Universiteit Brussel, Brussels, Belgium; 9 APC Microbiome Ireland and School of Microbiology, University College Cork, Cork, Ireland; 10 Advanced Green Energy and Environment Institute, Handong Global University, Pohang, Gyeongbuk South Korea; 11 Department of Bioscience Engineering, University of Antwerp, Antwerp, Belgium; 12 Department of Family Medicine, Georgetown University, Washington, DC USA; 13 Lawson Health Research Institute, and Departments of Microbiology & Immunology and Surgery, University of Western Ontario, London, Ontario Canada; 14 Department of Biology, Tufts University, Medford, MA USA; 15 Department of Food Science and Technology, University of Nebraska - Lincoln, Lincoln, NE USA

**Keywords:** Microbiome, Colon

## Abstract

An expert panel was convened in September 2019 by The International Scientific Association for Probiotics and Prebiotics (ISAPP) to develop a definition for fermented foods and to describe their role in the human diet. Although these foods have been consumed for thousands of years, they are receiving increased attention among biologists, nutritionists, technologists, clinicians and consumers. Despite this interest, inconsistencies related to the use of the term ‘fermented’ led the panel to define fermented foods and beverages as “foods made through desired microbial growth and enzymatic conversions of food components”. This definition, encompassing the many varieties of fermented foods, is intended to clarify what is (and is not) a fermented food. The distinction between fermented foods and probiotics is further clarified. The panel also addressed the current state of knowledge on the safety, risks and health benefits, including an assessment of the nutritional attributes and a mechanistic rationale for how fermented foods could improve gastrointestinal and general health. The latest advancements in our understanding of the microbial ecology and systems biology of these foods were discussed. Finally, the panel reviewed how fermented foods are regulated and discussed efforts to include them as a separate category in national dietary guidelines.

## Abstract (web summary)

Although fermented foods have been consumed for thousands of years, a clear definition has been lacking. This Consensus Statement outlines a definition for the term ‘fermented foods’ as determined by an expert panel convened by the International Scientific Association for Probiotics and Prebiotics in September 2019.

## Introduction

Fermented foods and beverages accompanied and likely facilitated the transition from hunter-gatherer communities to sessile agricultural communities in the Neolithic revolution about 14,000 years ago^1,2^. They have remained staples of human diets for centuries and are an increasingly popular food category. Yet, their emergent popularity in the past 20 years has led to numerous misunderstandings and questions. What constitutes fermentation? Do fermented foods necessarily contain live microorganisms? Are fermented foods the same as probiotic foods? Do microorganisms in fermented foods become established in the gut or influence the gut microbiota? Do fermented foods provide health benefits and, if so, how?

Accordingly, the International Scientific Association for Probiotics and Prebiotics (ISAPP) organized a meeting of clinical and scientific experts in family medicine, microbiology, food science and technology, ecology, immunology, and microbial genetics held in September 2019 to develop a consensus report on fermented foods (a category that includes fermented beverages). The main goals of this Consensus Statement are to provide researchers, health-care providers, industry, regulators and consumers with a clear and concise definition of fermented foods, to differentiate between fermented foods and probiotics, and to summarize what is known about the health effects and safety of fermented foods. This Consensus Statement also discusses the mechanistic rationale for how fermented foods could improve gastrointestinal and systemic health, the advancements in knowledge on the microbial ecology and systems biology of those foods, and the current regulatory considerations and position of these foods in dietary guidelines.

## Methods

The consensus panel was organized under the auspices of ISAPP, which is a non-profit organization governed by a volunteer board of directors. Although funded by member companies, ISAPP’s activities are not stipulated by industry. The mission is to provide objective, science-based information on probiotics, prebiotics and related health topics. Panel members were identified and invited based on their subject matter expertise and experience. An outline was developed and each expert was asked to address specific topics. The panel discussed each issue until consensus was reached. Following the meeting, each panellist wrote relevant sections and the assembled draft was reviewed and approved by all authors. The authors thank members of the ISAPP board of directors who did not directly participate in this consensus panel but who reviewed, provided comments and approved this manuscript: G. Gibson, E. Quigley, S. Salminen, K. Scott and H. Szajewska.

## Historical context

Humans must have learned early in their history that fermentation provided many important advantages for managing precious food resources. Fermentation can improve the functional properties of agricultural crops and transform bland raw materials into nutritious, palatable or intoxicating products. Certainly, fermentation would have been regarded as one of the most effective ways to preserve foods owing, in part, to the formation of organic acids, alcohols, bacteriocins and other antimicrobial end-products as a result of fermentation microorganisms^3^. Fermentation-associated microorganisms usually out-compete potential pathogenic and spoilage organisms, further enhancing food safety and stability. In the absence of potable water, fermented beverages, such as beer, wine, sour milk and cereal gruels, provided a safe and transportable source of liquids^4^. These qualities, along with the fermentation-mediated transformation of perishable raw food materials into organoleptically satisfying products, led to their adoption by nearly every culture worldwide.

One particular example of how fermented foods and human culture co-evolved is through dairy fermentations^5^. The consumption of fermented milk products, including cheese, pre-date human lactase persistence, suggesting that lactose removal might have been one of the initial aims of this process^6^. Similarly, the human attraction to flavour-potentiating nucleotides and amino acids that are enriched in certain fermented foods, such as soy sauce and miso, could have evolved as a result of the safety and nutritional benefits of those foods in early human diets^7^. The extended shelf-life of fermented foods and the removal of noxious plant compounds by fermentation still serve critical purposes in regions of the world that have low food security and poor access to refrigeration, electricity and clean water. Even in societies for which sanitation and preservation are not a problem, fermented foods constitute an important part of the human diet. It is estimated that more than 5,000 varieties of fermented foods (and beverages) are currently produced and consumed globally^8^.

Beyond their importance to public health and food preservation and quality, current epidemiological evidence suggests that diets rich in fermented foods can reduce disease risk and enhance longevity, health, and quality of life^9–11^. Nonetheless, with the exception of yoghurt and other cultured dairy products, few well-designed, randomized controlled trials (RCTs) on the health benefits of the array of fermented foods have been published. Likewise, hypothesis-driven research describing the mechanisms of how fermented foods affect human physiology is limited. Defining these gaps can provide a basis for future research, including experiments aimed at understanding the potential health benefits of fermented foods.

## Defining fermentation

Biochemists define fermentation as “an ATP-generating process in which organic compounds act as both donors and acceptors of electrons”^12^. Although this definition might be relevant for anaerobic lactic and ethanolic fermentations^13^ that occur in yoghurt, kimchi or wine, it does not apply to numerous other food fermentations. Fermentation as applied to foods and beverages has a much broader meaning and includes reactions and pathways that do not involve any of the criteria implicit in the strict biochemical definition. For example, aerobic metabolism is used by fungi responsible for koji, the starting material for soy sauce and miso, and in the manufacture of vinegar and kombucha by acetic acid bacteria (AAB)^14,15^. Accordingly, the panel proposes a broader definition that accounts for these variations in metabolic pathways. Thus, we define fermented foods and beverages as: “foods made through desired microbial growth and enzymatic conversions of food components”.

The definition requires the activity of microorganisms. Although endogenous or exogenous enzymes from plants, animals or other sources might be present, the activities of those enzymes alone are insufficient for a food to be regarded as fermented. This definition is sufficiently broad to include not only the fermentations noted earlier but also to distinguish fermentation from its microbiological converse, namely food spoilage. Whereas both processes occur via microbial growth and enzymatic activity on food constituents, spoilage is clearly unintentional and fermentation is deliberate and controlled to generate the desirable attributes.

### What is included or excluded in the fermented foods definition?

This definition of fermented foods and beverages accommodates the many products made globally from diverse starting materials (Box 1). The definition includes foods and beverages that are produced by fermentation but might not have living microorganisms at the time of consumption. Fermented foods, such as leavened breads, are baked after fermentation, effectively killing the fermentation microorganisms. The manufacture of some fermented foods (for example, most beers and wines) includes steps to remove live microorganisms from finished products. Although microbial inactivation or removal is not common to all fermentation processes, these products still qualify as fermented foods.

Some salad dressing, mustard and other condiments might include ingredients made by fermentation such as vinegar or sour cream. In our view, these foods would not satisfy the definition of a fermented food, even if they contained an appreciable amount of a fermented ingredient (Box 2), nor would a non-fermented food supplemented with added microorganisms be considered fermented. Lastly, there are chemically derived versions of fermented foods; these foods are not fermented (Box 2). For example, some soft cheeses can be made by chemical acidification and fruits and vegetables are often preserved by ‘pickling’ processes that do not require the presence of live microorganisms. In some regions, the production of so-called synthetic vinegar and non-brewed soy sauce use chemical processes^14,16^. Of note, some cured meat products (made with nitrate or nitrite salts) can be fermented or non-fermented.

Box 1 Fermented food classification based on the presence of live microorganisms **Fermented**

***Live microorganisms present***

- Yoghurt
- Sour cream
- Kefir
- Most cheeses
- Miso
- Natto
- Tempeh
- Non-heated fermented vegetables
- Non-heated salami, pepperoni and other fermented sausages
- Boza, bushera and other fermented cereals
- Most kombuchas
- Some beers

***Live microorganisms absent***

- Bread
- Heat-treated or pasteurized fermented vegetables, sausage, soy sauce, vinegar and some kombuchas
- Wine, most beers and distilled spirits
- Coffee and chocolate beans (after roasting)

**Not fermented**

- Chemically leavened bread
- Fresh sausage
- Vegetables pickled in brine and/or vinegar
- Chemically produced soy sauce
- Salted or cured processed meats and fish

Box 2 Key conclusions of this consensus paper

- Fermented foods are defined as foods made through desired microbial growth and enzymatic conversions of food components.
- Microorganisms (either autochthonous or intentionally added) determine the course and outcome of fermentation processes and contribute to the development of the characteristic properties of the final fermented food.
- Fermented food products should only be labelled as ‘containing probiotics’ when there is evidence that their live microbial components provide health benefits and the precise microbiological content is defined.
- A modern understanding of patterns of microbial community succession during the fermentation and ageing of fermented foods is being obtained through the application of metagenomics, metatranscriptomics and metabolomics.
- A better understanding of the health effects of fermented foods based on data available from population-based diet and health studies as well as new randomized controlled trials are needed to clarify the role of the consumption of fermented foods and of the live microorganisms they might contain in human health.
- When properly made, fermented foods and the bacteria and fungi responsible for their manufacture have a long history of safe use.
- Fermented foods could benefit health through the nutritive alteration of the ingredients, modulation of the immune system, the presence of bioactive compounds that affect intestinal and systemic function, or by modulating gut microbiota composition and activity.

### What is the difference between fermented foods and probiotics?

Fermented foods and beverages are sometimes characterized or labelled as “probiotic foods” or “contains probiotics”. These declarations might reflect efforts by manufacturers to communicate to consumers that living, health-promoting microorganisms are present in the product. However, as noted in a previous consensus statement^17^, the term ‘probiotic’ should only be used when there is a demonstrated health benefit conferred by well-defined and characterized live microorganisms. The health benefit must, at least in part, be due to the live microorganisms and must extend beyond any nutritional benefit of the food matrix. For these reasons, the terms ‘fermented food’ and ‘probiotics’ cannot be used interchangeably (Table 1).

**Table 1:** Distinctions between probiotics, fermented foods and probiotic fermented foods

					Microbial composition		
Probiotic substance	Definition	Format	Evidence for health benefit	Claim that is consistent with category^a^	Alive and present in levels demonstrated to provide benefit	Taxonomically defined to strain level	Genome sequence available
Probiotic	Live microorganisms that, when administered in adequate amounts, confer a health benefit on the host	No specific format required	Required	“Probiotic” can be used on the label along with a health benefit claim, such as “helps to reinforce the body’s natural defences”, if the claim is supported by evidence	Required	Required	Required
Fermented food	Foods made through desired microbial growth and enzymatic conversions of food components	Food	Not required	If live microorganisms are not present: “Foods made by fermentation”; if live microorganisms are present: “Contains live and active cultures”	Not required	Not required	Not required
Probiotic fermented food	Food fermented by or containing probiotic(s) with strain-specific evidence	Food	Required	Same as for probiotic	Required for probiotic but not for fermentation microorganisms	Required for probiotic but not for fermentation microorganisms	Required for probiotic but not for fermentation microorganisms
	Food fermented by or containing probiotic(s) without strain-specific evidence	Food	Required	“Contains probiotics”	Required for probiotic but not for fermentation microorganisms	Required for probiotic but not for fermentation microorganisms	Required for probiotic but not for fermentation microorganisms

^a^As allowed by local or regional regulations.

To label a product as a probiotic fermented food with an additional stipulated health benefit, evidence of a strain-specific benefit from a well-controlled intervention study is required together with proven safety and confirmation of sufficient numbers of that strain in the final product to confer the claimed benefit (Table 1). For example, traditional, spontaneously fermented sauerkrauts likely contain multiple strains of *Lactiplantibacillus plantarum* (previously *Lactobacillus plantarum*), but these uncharacterized and unidentified strains, at unknown doses, would not qualify as probiotics. By contrast, if *L. plantarum* 299v, a genetically characterized strain with clinically demonstrated probiotic properties^18,19^, was present at an efficacious dose until the end of shelf-life and there were no indications for inhibitory interactions of the sauerkraut matrix, this sauerkraut would meet the minimum criteria for a probiotic fermented food. Such products could contain an appropriately worded claim, for example, “probiotic sauerkraut containing *L. plantarum* 299v might improve intestinal well-being”, provided that local regulatory requirements are satisfied (Table 1).

In the absence of strain-specific evidence of a health benefit for the live microorganisms in a fermented food, some fermented foods could be appropriately labelled as “contains probiotics” (Table 1). This statement is only supported if at least one of the strains in the food meets the criteria implicit in the term probiotic and if the strain is a member of a well-studied species known to confer probiotic health benefits via the principle of ‘shared benefits’. This principle is based on the knowledge that certain bacterial species that are consistently active in human studies have conserved, or core, properties associated with improving health^20^. According to Hill et al.^17^ and Sanders et al.^20^, these bacterial species are sufficiently well studied such that most strains of that species can be reasonably expected to confer a health benefit. Consistent with this view, certain jurisdictions recognize several common species for which the term ‘probiotic’ can be used in foods. For example, Health Canada recognizes more than 20 species of the *Lactobacillus* genus complex and *Bifidobacterium* provided they are delivered at a minimum of 10^9^ colony-forming units per serving^21^. In Europe, health claims related to live yoghurt cultures and improved lactose digestion are approved by the European Food and Safety Authority based on the core presence of the lactase enzyme in yoghurt cultures (*Lactobacillus delbrueckii* subsp. *bulgaricus* and *Streptococcus thermophilus*)^22^. However, in our view, even if the fermented food contains one or more of those species, the label “contains probiotics” should only be used when the strains in the fermented food are defined to the strain level, the genome sequences are known and the strains are present at an appropriate number during product shelf-life (Table 1).

It is expected that the majority of fermented foods sold commercially today do not belong in the “probiotic fermented food” category. Instead, fermented foods and beverages often contain undefined microbial consortia, usually at variable levels, and their potential health benefits have generally not been demonstrated^23,24^. Thus, we affirm the suggestion from Hill et al.^17^ that manufacturers should state only that their product contains “live and active cultures” provided the food is not processed to remove or kill the fermentation microorganisms and that these microorganisms are present at levels that are expected for foods of that type (Table 1). For pasteurized fermented foods without live microorganisms in the final product, it is acceptable to label those foods as “foods made by fermentation” (Table 1). Even when characterized cultures are used to perform fermentations and are understood at the strain level, those microorganisms are mostly selected based on performance characteristics, such as rapid acidification, substrate conversion, and flavour and texture properties, rather than on health-related functions. In the absence of evidence for species-level ‘shared benefits’ and knowledge that the strains are present at an appropriate number during product shelf-life, we suggest that manufacturers consider other labelling options (as noted earlier).

### Do fermented foods contain prebiotics?

The presence of prebiotics, substrates selectively utilized by host microorganisms that confer a health benefit^25^, has been reported for several fermented foods and beverages. These examples would include fermented grains or vegetables^26^ as well as beer and wine^27,28^ that contain β-glucans, oligosaccharides and polyphenolic compounds^29^. Other fermented foods might contain prebiotics synthesized in situ by fermentation-associated microorganisms. For example, exopolysaccharides with prebiotic activity can be formed during dairy and cereal fermentations^30^. It is also possible that some fermented foods and beverages can contain both live microorganisms and prebiotic substrates. However, such products would not qualify as synbiotic foods^31^ in the absence of a demonstrated health benefit.

## Making fermented foods

### Which microorganisms are needed to make fermented foods?

To understand the scope of fermented foods in nutrition and health, it is necessary to acknowledge the wide diversity of microorganisms used for fermented food production. The most common fermented foods and beverages require lactic acid bacteria (LAB), AAB, bacilli or other bacteria, yeasts, or filamentous fungi. These microorganisms were among the very first to be isolated and characterized by Pasteur, Lister and other early microbiologists^32–34^ and have long served as model organisms in biology^35,36^ and as a source of industrial chemicals and bioactive molecules^37,38^. More recently, they were integral to the discovery and application of CRISPR technology^39^.

LAB are a group of Gram-positive, non-spore forming, aerotolerant bacteria that are phylogenetically positioned within the Firmicutes phylum, predominantly in the order Lactobacillales. They are among the most important and widely used microorganisms in food fermentations, serving essential functions in fermented dairy, meat, cereal and vegetable products^40^. LAB include the reclassified members of the Lactobacillaceae or *Lactobacillus* genus complex^41^ and numerous other taxa, including species of *Lactococcus* and *Tetragenococcus* associated with milk and soy sauce fermentations, respectively. Besides LAB, particular species of *Bacillus* and AAB are solely responsible for some fermented foods (for example, *Bacillus subtilis* used for natto, made from whole soybeans, and AAB for vinegar) or have important supporting roles as is the case for *Staphylococcus*, *Enterococcus*, *Brevibacterium* and *Propionibacterium* in sausage and cheese fermentations^42,43^. Among the fungi, ethanol-producing yeasts, usually species of *Saccharomyces*, are used for bread, beer, wine and various alcoholic fermentations. Interestingly, the domestication of *Saccharomyces cerevisiae* strains and their adaptation to a range of fermentation substrates and environments has led to the formation of distinct lineages associated with particular products^44,45^.

Similar domestication events are also likely responsible for the widespread use of atoxigenic filamentous fungi^46^. *Penicillium*, *Aspergillus* and *Rhizopus* are among the moulds commonly used for fermented dairy, meat and soy products and include proteinase, lipase and amylase-producing strains^47^. As described later, many food fermentations involve microbial communities consisting of multiple genera and taxa.

Considerable progress has been made towards understanding the function of individual microorganisms in fermented food production and then using that information to improve products and strains. Phylogenomic analyses have shown that, despite their general biochemical and physiological similarities, wine, beer and bread yeast strains evolved independently based on habitat and geography as well as through human-driven domestication^48,49^. Since the twentieth century, pure starter cultures have been developed to provide consistency and convenience and to accommodate large-scale industrial fermentations^50^. Typically, only one or two microbial strains (for example, bread, yoghurt, cheese) are necessary to initiate those fermentations^51^. Although technological performance properties remain one of the main criteria, the isolation and development of new strains increasingly relies on relevant genomic information and on the application of available molecular tools^50,52–54^.

**Fig. 1:**
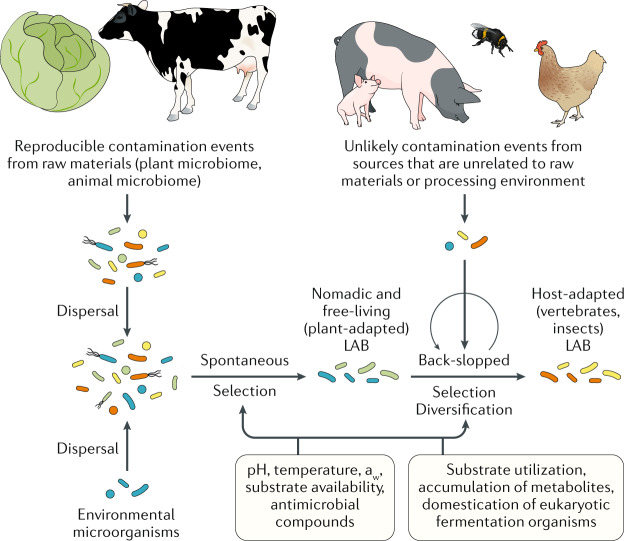
Processes that determine community assembly in traditional fermented foods. The conditions established during traditional and industrial fermentations provide a basis for controlling and manipulating autochthonous and allochthonous microorganisms. Microbial communities in spontaneous food fermentations are determined by dispersal and selection. In most spontaneously fermented foods, plant-associated or animal-associated microorganisms are dominant. Back-slopping of fermented foods eliminates dispersal limitation, and selection is the major principle that determines community assembly. Among lactic acid bacteria (LAB), nomadic and free-living species are dominant in spontaneous food fermentations while host-adapted species dominate many back-slopped fermentations^184^. Speciation and domestication have been demonstrated for eukaryotic food fermenting organisms, including *Saccharomyces cerevisiae* and *Aspergillus oryzae*, but not for bacteria^49,97^. If comparable raw materials and fermentation protocols are employed, community assembly in fermented foods is reproducible at the genus level (spontaneous food fermentations) or even at the species level (back-slopped food fermentations). The assignment of lifestyles to food-fermenting lactobacilli has been previously described^185^. a_w_, water activity.

Culture-dependent methods remain the gold standard for the strain-level characterization of fermentation microbiota; however, these methods are increasingly complemented by holistic, meta-omics methods (metagenomics, metatranscriptomics, metaproteomics and metabolomics)^55^. Molecular approaches have shown that fermented foods are frequently dependent on complex, multi-kingdom, microbial communities functioning in concert via dynamic succession processes^56–59^. However, despite this complexity, the presence of a so-called core microbiota (defined as widespread microorganisms that are central to the functions of these ecosystems) are often apparent in a wide range of fermented foods^60–64^. Provided that the starting materials are generally the same, spontaneous fermentations (relying on autochthonous or resident microorganisms present in the ingredients and/or surrounding environment) typically result in products that contain very similar microorganisms (even the same species), regardless of provenance^65^. For example, fermentations of cabbage and other green leafy vegetables are all initiated by *Leuconostoc mesenteroides* followed by *Lactiplantibacillus* species and *Levilactobacillus brevis*, independent of whether the product is called sauerkraut (Europe and North America), kimchi (Korea), suan-cai (China) or sinki (Nepal)^66^. This highly reproducible succession of fermentation microbiota in spontaneous vegetable fermentations, whereby the assembly of fermentation microbiota is limited by dispersal, reflects the stable association of these organisms with the raw materials (Fig. 1). Similar reproducible successions occur in fungi-fermented foods^67^. Collectively, these and other observations suggest that selective and competitive pressures drive microbiome assembly and succession dynamics and provide a basis for predicting the outcome of food fermentations^65^. Thus, provided that the raw materials and environmental conditions are consistent with the typical practices used for making that food and that salt concentrations, pH, atmosphere or other expected control measures are in place, unpredictable events, which constitute fermentation failure, are relatively rare (Fig. 1). In the absence of those conditions and control measures, food fermentations could result in inferior or unsafe products.

### What processes are involved in making fermented foods

The outcome of a food or beverage fermentation process depends on the microorganism-led conversion of substrates into metabolites that support the aroma and taste, appearance, preservation, and nutritional properties of the finished product. These characteristics are time-dependent and determined by the microbiota as well as by a range of physicochemical parameters, including temperature, pH, water activity, oxidation–reduction potential and substrate availability. How these intrinsic and extrinsic environmental parameters are ultimately managed can have profound effects on the final properties and characteristics of fermented foods^68^.

Systems and evolutionary biology approaches are now providing a rational basis for controlling or managing microbial diversity and community structure to achieve different fermentation processes^65,69^. Although fermented foods have long been studied as model systems to understand microbial ecology^70^, these latest efforts integrate broader ecological and evolutionary principles, including dispersal, selection, drift and diversification^65,71–73^. The contribution of these principles to community assembly in fermented foods is outlined in Fig. 1. The use of these principles enables the control of fermentation microbiota in food independent of whether the fermentation is initiated with starter cultures, spontaneously or by inoculation from a prior successful fermentation of the same type (that is, back-slopping) (Fig. 1). The application of systems biology approaches combined with community reconstructions can identify specific microbial interactions that drive community composition^51,74^, determine a genetic basis for particular microorganisms to live in a fermented food environment^75^ and recreate the domestication processes that generated the industrial cultures used in fermentations^76,77^. Ultimately, findings from those studies will help to address product variation and quality issues that occur even when starter cultures are used. They might also lead to the identification of biomarkers to monitor these foods throughout production and to predict nutritional and health-impacting qualities.

## Fermentation and food safety

### Does fermentation improve food safety?

Fermented foods that contain appreciable levels of fermentation-produced organic acids (>100 mM), combined with low water activity, salt, nitrite and other antimicrobials, have a long record of food safety^78^. Likewise, beverages containing 4% or more alcohol and pH values less than 4.5 are also considered microbiologically safe^79^. Many LAB, whether part of the autochthonous microbiota or added as starter cultures, are known to produce bacteriocins that inhibit undesirable bacteria, including *Listeria*, *Staphylococcus* and *Clostridium*^80^.

Food fermentations can also enhance food safety and nutritional quality by removing toxic or anti-nutritive compounds from the raw ingredients. For example, the removal of toxic compounds is a prominent feature of cereal, legume and tuber fermentations^81^. Bitter cassava, for example, contains cyanogenic glycosides that must be removed by fermentation, soaking or other suitable processes to avoid acute toxicity when consumed^82^. During sourdough fermentations, some LAB facilitate the degradation of phytate, a cereal grain-associated compound that chelates divalent cations and prevents their absorption in the gastrointestinal tract^83^. Reducing phytate results in enhanced calcium, magnesium, iron and zinc bioavailability from these breads^84–86^. Sourdough fermentation is also hypothesized to reduce the concentration of other immune-reactive proteins, including the amylase-trypsin inhibitor in wheat, and could therefore be better tolerated than conventional breads by individuals with non-coeliac wheat intolerance or irritable bowel syndrome^87^.

### Do fermented foods have food safety risks?

For any food product, there are safety concerns associated with live pathogenic microorganisms as well as toxins or metabolic products that can produce harmful effects. With few exceptions, food-fermenting LAB, yeasts and filamentous fungi are non-pathogenic and do not produce toxins or harmful end-products^88^. When properly made from safe and wholesome ingredients, fermented foods are rarely associated with gastroenteritis. Nonetheless, some cheeses and low-acid fermented foods can pose a safety risk if the food is contaminated with *Listeria monocytogenes*, *Salmonella*, *Clostridium botulinum* or other foodborne pathogens^89^. Although not a direct effect on safety, some microorganisms, including species of the Lactobacillaceae as well as *Enterococcus* and *Staphylococcus* associated with long-ripened cheeses, sausages and other fermented foods, can carry transmissible antibiotic-resistance genes^90–92^.

The microbial metabolites of some fermented foods can, under certain circumstances, also present safety risks. Alcohol (for example, wine, beer and liquor) and salt (for example, soy sauce or kimchi) are inherent constituents of some fermented foods and should be consumed in moderation. Histamine, tyramine and other biogenic amines are formed by some LAB via the decarboxylation of amino acids during the fermentation of cheese, meats, vegetables, soybeans and wine^93^. In the absence of host-mediated detoxification systems, these amines can cause mild to more severe effects such as migraines^94^. Several strategies have been adopted to reduce or mitigate biogenic amine formation, including hygiene to minimize the occurrence of microorganisms producing these compounds and using decarboxylase-negative starter cultures^95,96^.

Mycotoxins are a potential concern for all fermented foods produced with filamentous fungi. However, domestication and careful strain selection have effectively eliminated mycotoxin-producing lineages of *Aspergillus* and *Penicillium* from koji, cheese and other fermented foods^76,97,98^. Other microbial metabolites, including citrulline and reuterin, are precursors of the toxic compounds ethyl carbamate^99^ and acrolein^100^, respectively. Both occur in alcoholic beverages as well as in other fermented foods. However, their risks to human health from the exposure to fermented foods have not been established^101,102^.

## Fermented foods and human health

### What is the current evidence that fermented foods benefit human health?

Consumer interest in fermented foods has been driven in large part by their suggested nutritional benefits, and this interest has led to renewed popularity of these foods on nearly every continent^24,103^. However, except for yoghurt and cultured dairy products, few human clinical studies have been performed to verify their benefits^23,24,104^. Yoghurt consumption is associated with reductions in adiposity factors (BMI, waist circumference)^105^, type 2 diabetes mellitus and cardiovascular disease (see reviews^106,107^), among other positive indications^108^. Although much of this evidence is based on prospective or epidemiological studies, more than 20 RCTs with yoghurt and cultured milk products have been reported for both healthy individuals and patient population groups^109^. Likewise, milk kefir^110^, kimchi^111^, sauerkraut^112^, natto^113^, vinegar^114^ and sourdough bread^115^ have been investigated in at least one RCT. By contrast, evidence of health promotion for other fermented foods (for example, kombucha) is mostly limited to chemical analyses and animal and cell culture models^24^.

A better understanding of the health benefits of fermented foods will be obtained from harvesting information from existing population-based diet and health databases as well as with new RCTs. These studies should address the health outcomes arising from the intake of differentiated fermented food categories (including fermented dairy products and other fermented foods with living versus dead microorganisms), food types (such as fermented vegetables, fermented soy and yoghurt), and individual fermented food products with well-characterized strains and nutrient compositions. Large, placebo-controlled RCTs will need to account for the known limitations of these types of nutrition study, including blinding, sample size, diet control, dietary recall and adequate intervention times, as well as the challenges specific to fermented foods (in particular, how to provide relevant placebo treatments). To prevent the foods from being easily distinguished by study participants, placebo controls might need to be made to provide the same sensory attributes expected for the fermented foods being tested. Retrospective cohort or, preferably, prospective cohort studies that meet the Bradford Hill criteria should be used^116^ and efforts should be made to avoid misleading or unwarranted conclusions^117^. It should be noted that additional challenges exist for cohort studies because dietary databases do not often include fermented foods as a category and critical aspects of those foods might not be reported (for example, percent fat, percent protein or microbiological content).

### What is the mechanistic basis for the health benefits of fermented food?

**Fig. 2:**
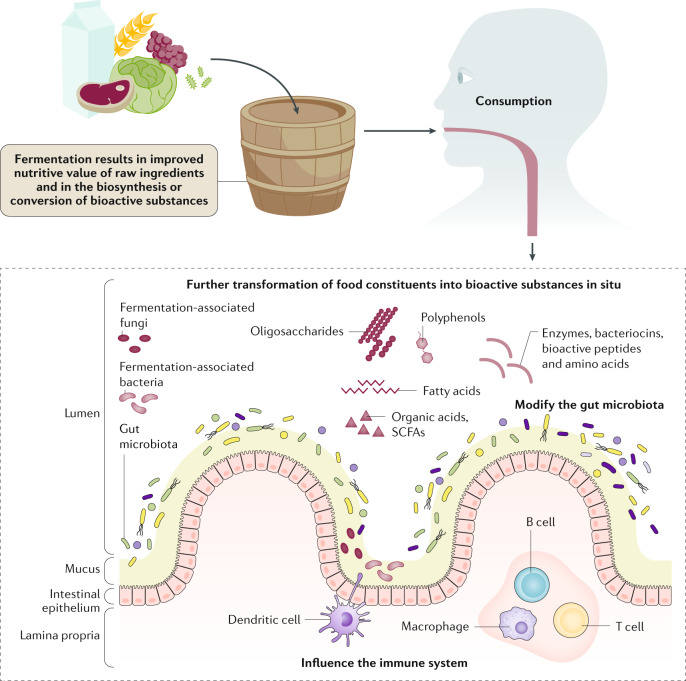
Mechanistic basis for the health benefits of fermented foods. Health benefits, beyond the nutritional contributions of the raw ingredients, result from the removal, synthesis and transformation of the food components during fermentation by the activities of fermentation-associated microorganisms. Such actions can result in improved nutritive value of the food (for example, through phytate detoxification or vitamin synthesis) or in the generation of biologically active compounds (for example, bioactive peptides or conjugated linoleic acid). Food constituents and fermentation products, along with any remaining viable fermentation microorganisms, are consumed and enter the intestinal tract. Those microorganisms, along with resident members of the gut microbiota, might further transform food constituents in vivo into bioactive substances such as peptides, bacteriocins, amino acids, conjugated linoleic acid or organic acids. The constituents of fermented foods and fermentation-associated microorganisms and their cell products can interact with gut microbiota, the intestinal epithelium or the host immune system. SCFAs, short-chain fatty acids.

Knowledge on the specific health-promoting properties of fermented foods provides a foundation to evaluate how those properties vary by food type, strain composition and production methods. Several routes for health promotion by fermented foods are proposed (Fig. 2), including nutritive alteration of raw ingredients and the biosynthesis of bioactive compounds, modification of the human gut microbiota, and development and modification of the immune system.

Microbial activity during food fermentations results in the enrichment and/or removal of compounds that affect the nutritional composition of the final food product^118^. Microorganisms reduce the concentrations of high-calorie monosaccharides and disaccharides (glucose, sucrose and fructose) present in milk, meat and plants via catabolic pathways. Reductions in certain sugars could also reduce the glycaemic index^119,120^ and improve food tolerability (for example, lactose in dairy foods, fructans in wheat, or raffinose, stachyose and verbascose in soybeans and legumes)^121^. Fermentation can result in the hydrolysis of polysaccharides, proteins or fats, thereby increasing their digestion^122–124^. Other enzymatic transformations with important nutritional implications also occur, including detoxification reactions (for example, degradation of linamarin in bitter cassava) and the removal of anti-nutritive factors (for example, inactivation of trypsin inhibitor in soybeans and phytic acid in cereals such as sorghum)^125–127^. For polyphenol-containing foods, the conversion of phenolic compounds by lactobacilli^128^ increases the bioavailability of flavonoids, tannins and other bioactive compounds^129,130^. The biosynthesis of vitamins, amino acid derivatives, organic acids and cofactors can also occur during fermentation^23,131,132^, with effects at either local gastrointestinal or systemic sites. Some of these compounds are broadly distributed between fermented food types (such as lactic acid^133^ and acetic acid^134^), whereas others are common in certain foods (for example, alkyl catechols^135^) or limited to certain microorganisms with specific enzymatic activities (for example, synthesis of γ-aminobutyric acid, conjugated linoleic acid or angiotensin-converting enzyme inhibitors^132^).

Multiple studies in humans have shown that microorganisms in fermented foods can survive gastric transit and reach the colon^112,136–144^. Indeed, many of the LAB that dominate lactic acid-fermented foods possess intrinsic characteristics that promote their ability to survive gastric transit (for example, acid and bile tolerance)^145^. Depending on individual dietary habits, fermented food-associated LAB can transiently constitute between 0.1% and 1% of the bacteria in the large intestine and a comparable proportion in the small intestine^145^. This percentage is based on current estimates of autochthonous microbiota in the gastrointestinal tract^146^ and the presence of up to 10^11^ LAB cells in a single serving of many fermented foods, such as yoghurt or kefir, that contain live and active microorganisms. Similarly, another study published in 2020 showed that food-associated LAB reached faecal metagenome abundances of >0.1%^147^. Although these microorganisms are unlikely to maintain long-term residence in the intestine, some fermented food microorganisms are known to be metabolically active in the gastrointestinal tract^144,148^, and short-term colonization could be sufficient to synthesize bioactive compounds, inhibit intestinal pathogens and mediate epithelial modulatory effects (for example, via interaction with Toll-like receptors^149^). Such interactions would be augmented by the repeated daily consumption of the fermented food. According to population-based studies and RCTs, fermented foods can also influence the composition of the gut microbiota^136,150–153^. Modulation of the gut microbiota can result from the living (or inactivated) microorganisms in those foods, the nutritional components and metabolites released as a result of fermentation, and changes these food constituents confer on the host immune system. These effects are likely dependent on inter-individual differences in host physiology and gut microbiota composition^154^.

As approximately 70% of the human immune system is located in the gastrointestinal tract^155^, foods and beverages are the major conduit of contact between external antigens and the human body. The gastrointestinal tract is vulnerable to the initial pattern of microbial colonization during the first months of life^156^, potentially setting a critical window for microbial stimuli effects on the immune system. In one cross-sectional study, fermented food intake (fermented vegetables) during early childhood was associated with a reduced risk of childhood atopy^157,158^. In another epidemiological study, fermented food consumption combined with common daily-life exposure (for example, hand versus machine dishwashing) also reduced the risk of childhood allergies^157,158^. The authors from the former study further reported that an anthroposophic lifestyle (low antibiotic use and vaccinations and high intake of fermented vegetables) was associated with differences in infant microbiome structure, including a higher abundance and diversity of LAB, and a higher concentration of acetate compared with infants from a traditional lifestyle^159^. Fermented food intake is also one of the synergistic factors associated with a farming upbringing, a lifestyle factor that has consistently been associated with reduced allergy and asthma risk (reviewed elsewhere^160^). These associations could indicate that a lack of fermented foods in modern, industrialized societies constitutes a substantial loss in exposure to non-harmful microorganisms important for immune system development and maintenance.

Although fermented foods such as milk kefir^161^ have been shown to modulate immune responses in numerous animal models, RCTs or prospective studies on the human immune system have yet to be performed. It is expected that the modulation of the human immune system by fermented foods would be the result of the combined effects of compounds present in the starting ingredients and those formed during fermentation as well as of living and dead or inactivated microorganisms. Those fermentation-associated microorganisms and their cell components (for example, peptidoglycan, surface proteins, exopolysaccharides and lipoteichoic acid) are already known to be immune reactive according to animal model and in vitro studies^149,162–164^. Knowledge about other immune-modulating compounds, such as d-phenyllactic acid, produced by lactic acid bacteria in situ^165^, is still emerging. Ultimately, the precise molecular stimuli in fermented foods responsible for immunomodulation probably depend on the total composition of the product^133,134,166^.

### What are the regulatory considerations for fermented foods?

Guidelines that govern food fermentation are covered in international regulations and are mainly concerned with food safety^167,168^. The use of microbial cultures is also regulated and includes criteria for establishing safety, such as the ‘Generally Recognized As Safe’ designation in the USA or the ‘Qualified Presumption of Safety’ list in Europe. The latter, for example, is a designation assigned by the European Food and Safety Authority to groups of microorganisms that, in general, do not raise safety concerns as components of foods, including fermented foods^169^. Strains developed by the use of recombinant DNA technology or those that are genetically modified have different regulatory controls. For example, in the USA, genetically modified strains must have a ‘Generally Recognized As Safe’ status, whereas in Europe, such products require Qualified Presumption of Safety status^170^.

The identification of core microbial components in fermented foods has the potential to lead to new regulations around the labelling of these foods. Regulations could be used to ensure that minimum requirements relating to the involvement of specific microbial taxa in the fermentation process are met. Only a few standards exist, mostly for cultured dairy products. For example, the Codex Alimentarius states that yoghurt should be made using a combination of *S. thermophilus* and *L. delbrueckii* subsp. *bulgaricus* and that kefir is a fermented milk consisting of *Lentilactobacillus kefiri* and species of the genera *Leuconostoc*, *Lactococcus* and *Acetobacter*, in addition to lactose-fermenting yeasts (*Kluyveromyces marxianus*) and non-lactose-fermenting yeasts (*Saccharomyces unisporus*, *S. cerevisiae* and *Saccharomyces exiguus*)^171^. Similar standards could emerge as the microorganisms present in other fermented foods are identified (for example, kombucha and water kefir).

### What is the standing of fermented foods in dietary guidelines?

Fermented foods are widely consumed around the world and have been estimated to account for approximately one-third of the human diet^172,173^. However, with few exceptions, fermented foods are generally absent as a recommended category in dietary guidelines^172,174,175^. The only country, to our knowledge, that has a specific guideline is India, which encourages pregnant women to consume fermented foods^176^. Other countries, including the USA and Canada, mention yoghurt and kefir in the dairy products section^136,138^, but there is no specific emphasis on fermented foods. Owing to the high levels of live, potentially health-promoting microorganisms in many fermented foods, these foods have been advocated for inclusion in dietary recommendations^132,172,174^. To advance this field, studies that collect dietary information should also track foods that contain live cultures. Adding granularity to dietary intake data so that fermented foods are not subsumed under other categories will enable researchers to better understand the role of these foods in health.

## Implications for stakeholders

One of the main goals of this panel was to bring scientific clarity to the rapidly growing field of fermented foods and beverages. We anticipate that the outcomes described in this report (Box 2) could affect a range of stakeholders, including consumers, industry, government, and science communicators.

### Consumers

Although consumers have become increasingly interested in fermented foods, it is unfortunate that, in our opinion, much information available on fermented foods in popular press magazines, websites and social media is exaggerated or inaccurate. For example, on the many internet and popular magazine lists of the ‘best super foods’, fermented foods are often ranked at the top. Such labels, while perhaps useful for marketing, do not convey accurate information for consumers regarding nutritional or other specific properties of fermented foods. Furthermore, as discussed earlier, fermented foods are frequently considered as probiotic foods, even when live microorganisms are absent in the final product and the health benefits have not been clinically demonstrated. This report clarifies these points for consumers and communicators.

### Industry

As noted previously, fermented foods and beverages were among the first processed foods. Bread, beer, wine and fermented dairy, soy and other products continue to represent a considerable portion of the total processed foods industry. This form of processing remains extremely important in many parts of the world, whereby fermented foods can enhance both food security and sustainability^177^. Food fermentation can also provide new strategies for industry to address contemporary socioeconomic and health challenges involving ageing, malnutrition and obesity^178^. Manufacturers who produce and market fermented foods can benefit from clear definitions and criteria for what constitutes probiotic fermented foods. In particular, we reaffirm the statement in Hill et al.^17^ that fermented foods are not equivalent to probiotic foods. Many fermented food products have no evidence that their live microbial component provides health benefits and the precise microbiological content is rarely defined. Without this level of characterization, they should not be labelled as “containing probiotics”. Some manufacturers supplement fermented foods with microorganisms after a heat treatment, perhaps to satisfy consumer interest in adding live microorganisms to their diet. These products, in our view, do not reflect the expected characteristics of fermented foods containing live microorganisms. In general, there is no expectation that fermented foods must contain live microorganisms. The most notable exception is for yoghurt, where, depending on the jurisdiction, specific requirements can exist. Industry is responsible for producing fermented foods following good manufacturing practices and should practice advertising and labelling that is truthful and informative and should be consistent with the criteria stipulated above.

### Government

In most jurisdictions, governments provide regulatory oversight of the safety and marketing of fermented foods, including advertising, product labelling and health benefit claims. In Europe, a broad range of fermented foods are made in accordance with so-called Protected Designation of Origin requirements that impose geographical, manufacturing and quality requirements^179^. Similar arrangements also exist in other countries. Although the Protected Designation of Origin framework is designed to control product claims about geographical origins and production practices and not microbiological properties of foods, per se, these protections can dictate the type or nature of the cultures used in cheese, sausages, bread, vinegar and other fermented foods. Thus, for these products, governments can indirectly influence how fermented products are produced as well as the safety and quality properties. This process is especially relevant as industrialization and high-throughput production practices have been adopted even by traditional small-scale manufacturers^180^.

Government agencies are also responsible for providing accurate and informative nutritional labelling and for reviewing and approving health benefit claims. However, as already noted, most regulatory agencies have not considered the potential inclusion of fermented foods in dietary guidance programmes beyond their nutritional contribution to health. Nonetheless, as more clinical and epidemiological studies are reported, such efforts could be warranted.

## Conclusions

For more than a century, microbiologists have sought to identify and describe the relevant ‘microbial parts’ within fermented foods and beverages. Only in the past two decades have researchers from multiple scientific disciplines, including systems and molecular biology, microbial ecology, and bioinformatics, begun to understand how those parts are assembled to build microbial communities that are ultimately responsible for the attributes associated with fermented foods and beverages. Collectively, this research provides a rational basis for improving both the functional characteristics and nutritional properties of these foods. It could also be feasible to identify and introduce novel microbial species that can augment desirable traits^50^. Many spontaneously fermented foods serve as a rich reservoir of potentially valuable strains^181–183^. Of particular interest is the possibility of predicting the quality attributes of fermented foods and beverages based on the initial microbial composition of the raw materials. Ultimately, the production of fermented foods and beverages with greater quality control will ensure the delivery of products that provide flavour, texture and health-related attributes.
